# Supplementary material for: The Genetic Diversity and Antimicrobial Resistance of Pyogenic Pathogens Isolated from Porcine Lymph Nodes
Source: Antibiotics (Basel). 2023 Jun 7;12(6):1026. doi: 10.3390/antibiotics12061026 (PMC10294850; doi:10.3390/antibiotics12061026)
Supplement: Supplementary file 1 [file antibiotics-12-01026-s001.zip › Table S1.pdf]

Table S1. Distribution of minimum inhibitory concentration (MIC) of eight antimicrobial agents; MIC_{50} and MIC_{90} values for the studied *Streptococcus* spp. isolates from pigs (*n*=48).

**Antimicrobial agent**[a]	**Number of Isolates with MIC (μg/mL)**[b]												**MIC_{50}**	**MIC_{90}**
	**0.003**	**0.004**	**0.006**	**0.008**	**0.012**	**<0.016**	**0.016**	**0.023**	**0.032**	**0.047**	**0.064**	**0.094**		
**PEN**	2	16	13	4	3		6	3		1			0.006	0.016
**AMC**						8	18	13	1	5	3		0.016	0.047
**CTX**					1		8	8	13	6	5	7	0.032	0.094
	**0.25**	**0.38**	**0.5**	**0.75**	**1**	**1.5**	**2**	**3**	**4**	**6**	**8**	**12**		
**CIP**	3	9	18	16	1		1						0.5	0.75
**GEN**			3	7	10	10	9	4	5				1.5	3
	**0.064**	**0.094**	**0.125**	**0.19**	**0.25**	**0.38**	**0.5**	**0.75**	**1**	**1.5**	**2**	**>256**		
**ERY**	1	6	11	6	4	5	2	9				4	0.19	0.75
	**0.125**	**0.19**	**0.25**	**0.38**	**0.5**	**0.75**	**1**	**1.5**	**3**	**8**	**12**	**>32**		
**SXT**	6	10	3	7	4	6	2	4	2	1		3	0.38	3
	**0.094**	**0.125**	**0.19**	**0.25**	**0.38**	**0.5**	**4**	**12**	**16**	**24**	**32**	**48**		
**DOX**	2	3	5	10	2	2	1	3	11	5	2	2	0.5	24

[a] PEN – penicillin, AMC – amoxicillin/clavulanic acid, CTX – cefotaxime, CIP – ciprofloxacin, ERY – erythromycin, DOX – doxycycline, GEN – gentamicin, SXT – trimethoprim-sulfamethoxazole; [b] MIC range for tested antimicrobial agents.
