# Supplementary material for: The Genetic Diversity and Antimicrobial Resistance of Pyogenic Pathogens Isolated from Porcine Lymph Nodes
Source: Antibiotics (Basel). 2023 Jun 7;12(6):1026. doi: 10.3390/antibiotics12061026 (PMC10294850; doi:10.3390/antibiotics12061026)
Supplement: Supplementary file 1 [file antibiotics-12-01026-s001.zip › Table S2.pdf]

Table S2. Distribution of minimum inhibitory concentration (MIC) of eight antimicrobial agents; MIC_{50} and MIC_{90} values for the studied *Rhodococcus equi* isolates from pigs (n=17).

**Antimicrobial agents**[a]	**Number of Isolates with MIC (µg/mL)**[b]												**MIC_{50}**	**MIC_{90}**
	**0.75**	**1**	**1.5**	**2**	**3**	**4**	**6**	**8**	**12**	**16**	**24**	**>32**		
**PEN**				2	1	3	2		1			8	12	>32
**AMC**	3	3	6	3	2								1.5	2
**CTX**						3	2	1				11	>32	>32
	**0.25**	**0.38**	**0.5**	**0.75**	**1**	**1.5**	**2**	**3**	**4**	**6**	**8**	**>32**		
**CIP**	1	4	1	4	4	2						1	0.75	1.5
**GEN**	1	6	4	5							1		0.5	0.75
	**0.125**	**0.19**	**0.25**	**0.38**	**0.5**	**0.75**	**1**	**1.5**	**2**	**3**	**4**	**6**		
**ERY**	2	5	4	4	2								0.25	0.38
	**0.5**	**0.75**	**1.5**	**2**	**3**	**4**	**6**	**8**	**12**	**16**	**24**	**>32**		
**SXT**	1		2	6			1	1	1			5	2	>32
	**1.5**	**2**	**3**	**4**	**6**	**8**	**12**	**16**	**24**	**32**	**48**	**>256**		
**DOX**	1			3	4	6	3						8	12

[a] PEN – penicillin, AMC – amoxicillin/clavulanic acid, CTX – cefotaxime, CIP – ciprofloxacin, ERY – erythromycin, DOX – doxycycline, GEN – gentamicin, SXT – trimethoprim-sulfamethoxazole; [b] MIC range for tested antimicrobial agents.
