# Supplementary material for: The Genetic Diversity and Antimicrobial Resistance of Pyogenic Pathogens Isolated from Porcine Lymph Nodes
Source: Antibiotics (Basel). 2023 Jun 7;12(6):1026. doi: 10.3390/antibiotics12061026 (PMC10294850; doi:10.3390/antibiotics12061026)
Supplement: Supplementary file 1 [file antibiotics-12-01026-s001.zip › Table S3.pdf]

Table S3. Distribution of minimum inhibitory concentration (MIC) of eight antimicrobial agents; MIC_{50} and MIC_{90} values for the studied *Staphylococcus aureus* isolates from pigs (n=5).

**Antimicrobial agents**[a]	**Number of Isolates with MIC (µg/mL)**[b]												**MIC_{50}**	**MIC_{90}**
	**0.023**	**0.032**	**0.047**	**0.064**	**0.094**	**0.125**	**0.19**	**0.25**	**0.38**	**0.5**	**0.75**	**1**		
**PEN**	1	2	1			1							0.032	0.125
**AMC**					1	3		1					0.125	0.25
**CIP**								4			1		0.25	0.75
**GEN**						2	3						0.19	0.19
**ERY**				2	2					1			0.094	0.5
**SXT**						2	1	1	1				0.19	0.38
**DOX**								1	3	1			0.38	0.5
	**0.75**	**1**	**1.5**	**2**	**3**	**4**	**6**	**8**	**12**	**16**	**24**	**>32**		
**CTX**					2	1			1			1	4	>32

[a] PEN – penicillin, AMC – amoxicillin/clavulanic acid, CTX – cefotaxime, CIP – ciprofloxacin, ERY – erythromycin, DOX – doxycycline, GEN – gentamicin, SXT – trimethoprim-sulfamethoxazole; [b] MIC range for tested antimicrobial agents.
