# Supplementary material for: The Genetic Diversity and Antimicrobial Resistance of Pyogenic Pathogens Isolated from Porcine Lymph Nodes
Source: Antibiotics (Basel). 2023 Jun 7;12(6):1026. doi: 10.3390/antibiotics12061026 (PMC10294850; doi:10.3390/antibiotics12061026)
Supplement: Supplementary file 1 [file antibiotics-12-01026-s001.zip › Figure S1.docx]

**
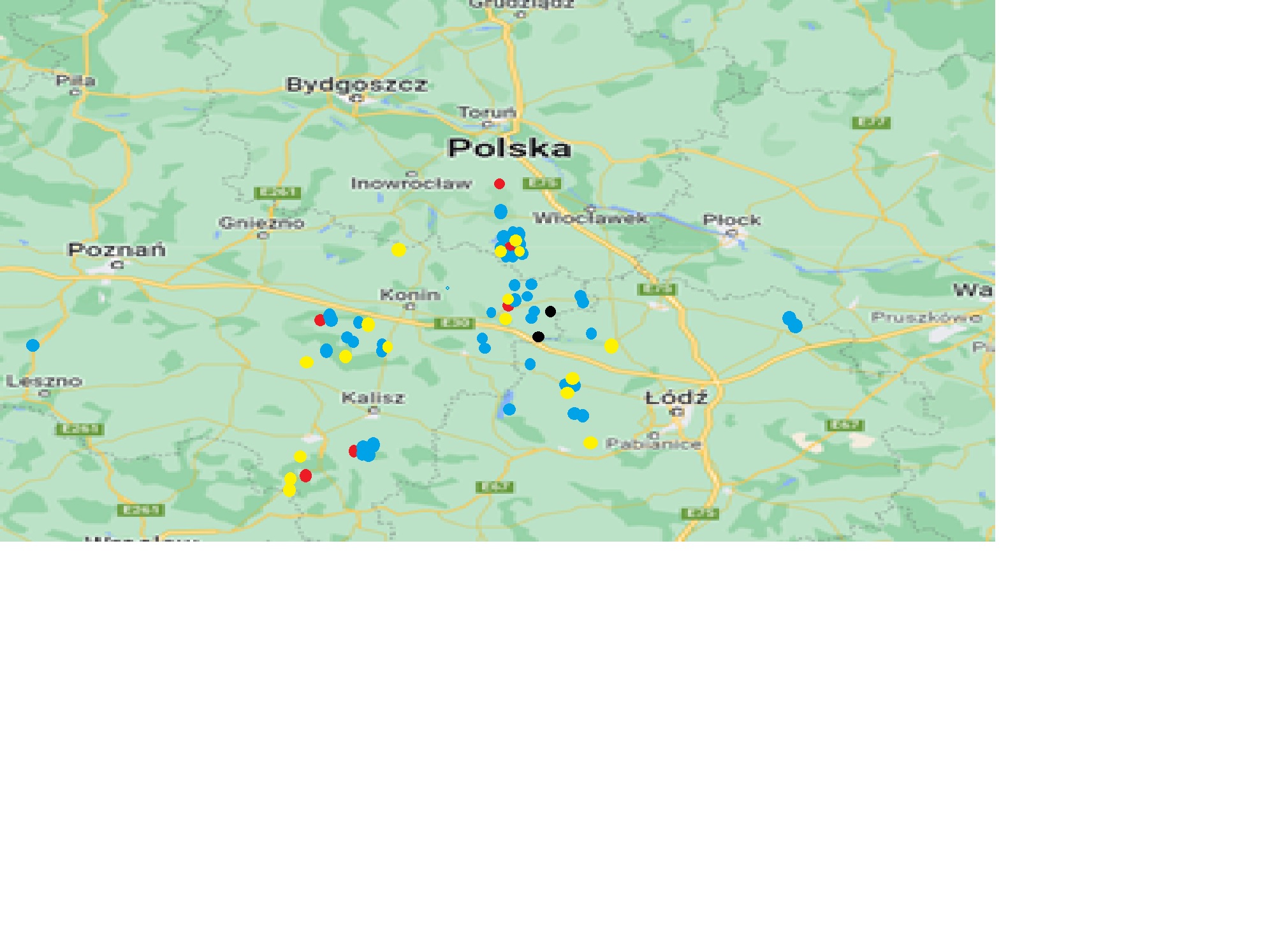
**

**Figure S1.** Location of slaughterhouses and farms of the studied pigs, from which pyogenic bacteria were isolated. Location of farms where pyogenic pathogens were isolated from pigs and location of slaughterhouses. Black dot—slaughterhouse; blue dot—*S. dysgalactiae*-positive farm; red dot—*S. aureus*-positive farm; yellow dot—*R. equi*-positive farm.
