# Supplementary figures and images for: The Genetic Diversity and Antimicrobial Resistance of Pyogenic Pathogens Isolated from Porcine Lymph Nodes
Source: Antibiotics (Basel). 2023 Jun 7;12(6):1026. doi: 10.3390/antibiotics12061026 (PMC10294850; doi:10.3390/antibiotics12061026)

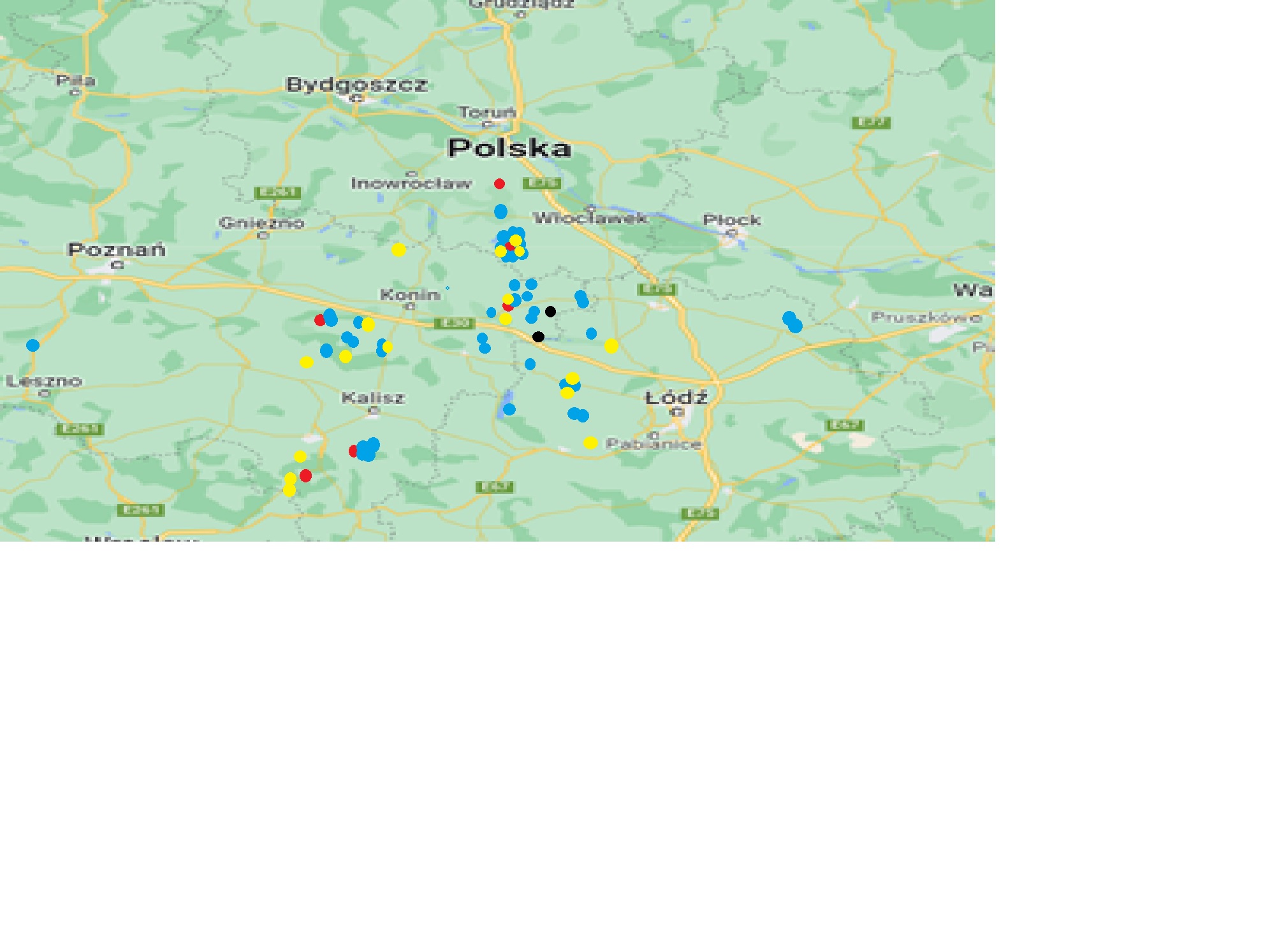

Supplement: Supplementary file 1 [file antibiotics-12-01026-s001.zip › Figure S1.jpg]

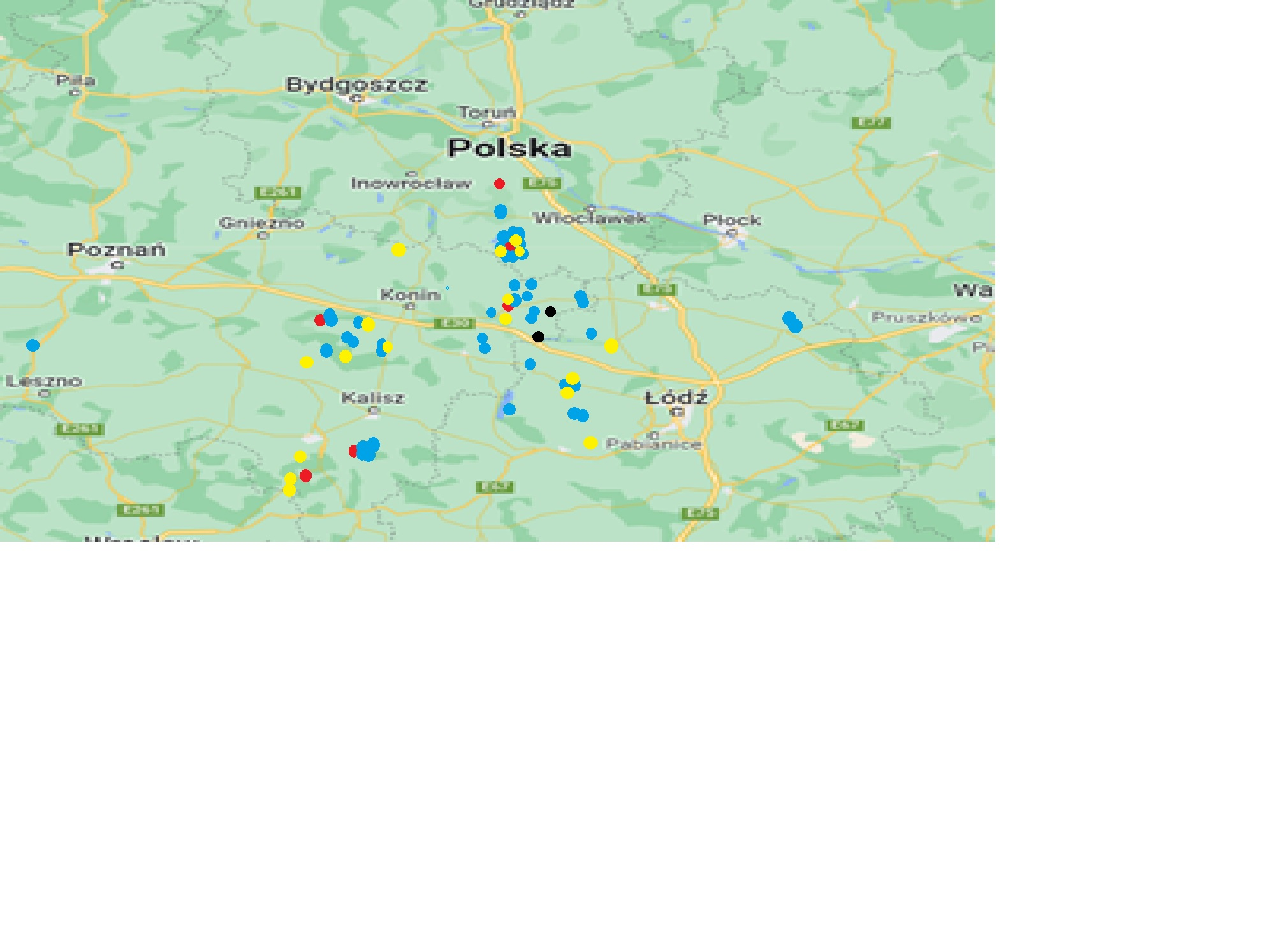

Supplement: Supplementary file 1 [file antibiotics-12-01026-s001.zip › Figure S1.tif]
